# ASSOCIATIONS BETWEEN NEIGHBORHOOD DISADVANTAGE AND MOBILITY LIMITATIONS AMONG AFRICAN AMERICANS

**DOI:** 10.1093/geroni/igac059.456

**Published:** 2022-12-20

**Authors:** Dextiny McCain, Adrienne Aiken Morgan, Alexa Allan, Alyssa Gamaldo, Regina Wright, Roland J Thorpe, Jr., Jason Allaire, Keith Whitfield

**Affiliations:** North Carolina Central University, Durham, North Carolina, United States; University of North Carolina at Chapel Hill, Chapel Hill, North Carolina, United States; The Pennsylvania State University, University Park, Pennsylvania, United States; Pennsylvania State University, State College, Pennsylvania, United States; University of Delaware, Newark, Delaware, United States; Johns Hopkins University, Baltimore, Maryland, United States; North Carolina State University, Raleigh, North Carolina, United States; University of Nevada, Las Vegas, Las Vegas, Nevada, United States

## Abstract

Research has shown that socioeconomic factors influence physical mobility; however, less is known about the influence of neighborhood disadvantage on mobility among African American older adults. Data from the Baltimore Study of Black Aging (N=602) were used to examine associations between neighborhood disadvantage, as measured by the Area Deprivation Index (ADI), and self-reported mobility limitations. Two-step logistic regression showed that participants residing in more disadvantaged neighborhoods, based on national ADI, had higher odds of mobility limitations, but those in less disadvantaged neighborhoods, based on state ADI, had higher odds of mobility limitations (national: OR = 1.02 (95% CI: 1.00–1.03); state: OR = 0.78 (95% CI: 0.65–0.92)). Stratified models showed the association was significant only within women (national: OR = 1.02 (95% CI: 1.00–1.04); state: OR = 0.77 (0.63-0.95)). These findings suggest that neighborhood disadvantage is an essential consideration when examining mobility limitations among African Americans.

